# Neuromyelitis Optica in Austria in 2011: To Bridge the Gap between Neuroepidemiological Research and Practice in a Study Population of 8.4 Million People

**DOI:** 10.1371/journal.pone.0079649

**Published:** 2013-11-05

**Authors:** Fahmy Aboul-Enein, Thomas Seifert-Held, Simone Mader, Bettina Kuenz, Andreas Lutterotti, Helmut Rauschka, Paulus Rommer, Fritz Leutmezer, Karl Vass, Agathe Flamm-Horak, Robert Stepansky, Wilfried Lang, Elisabeth Fertl, Thomas Schlager, Thomas Heller, Christian Eggers, Georg Safoschnik, Siegrid Fuchs, Jörg Kraus, Hamid Assar, Stefan Guggenberger, Martin Reisz, Peter Schnabl, Martina Komposch, Philipp Simschitz, Alena Skrobal, Alexander Moser, Mario Jeschow, Dorothea Stadlbauer, Manfred Freimüller, Michael Guger, Susanne Schmidegg, Claudia Franta, Vera Weiser, Stefan Koppi, Margret Niederkorn-Duft, Bettina Raber, Iris Schmeissner, Julia Jecel, Alexander Tinchon, Maria K. Storch, Markus Reindl, Thomas Berger, Wolfgang Kristoferitsch

**Affiliations:** 1 Department of Neurology, Sozialmedizinisches Zentrum Ost Donauspital, Vienna, Austria; 2 Department of Neurology, Karl Landsteiner Institute for Neuroimmunological and Neurodegenerative Disorders, Vienna, Austria; 3 Department of Neurology, Medical University Graz, Graz, Austria; 4 Clinical Department of Neurology, Innsbruck Medical University, Innsbruck, Austria; 5 Department of Neurology, Medical University of Vienna, Vienna, Austria; 6 Department of Neurology, Krankenhaus der Barmherzigen Brüder, Vienna, Austria; 7 Department of Neurology, Krankenanstalt Rudolfstiftung, Vienna, Austria; 8 Department of Neurology, Konventspital Barmherzige Brüder, Linz, Austria; 9 Department of Neurology, Krankenhaus Hietzing mit Neurologischem Zentrum Rosenhügel, Vienna, Austria; 10 Department of Neurology, Paracelsus Medical University, Salzburg, Austria; 11 Department of Neurology, Landes-Nervenklinik Wagner-Jauregg, Linz, Austria; 12 Department of Neurology, Landeskrankenhaus Steyr, Steyr, Austria; 13 Department of Neurology, Private Hospital Mariahilf Humanomed Hospital, Klagenfurt, Austria; 14 Department of Neurology, Klinikum Klagenfurt, Klagenfurt, Austria; 15 Department of Neurology, Landesklinikum Horn, Horn, Austria; 16 Department of Neurology, Neurologisches Therapiezentrum Kapfenberg, Kapfenberg, Austria; 17 Department of Neurology, Krankenhaus der Barmherzigen Schwestern Ried, Ried, Austria; 18 Department of Neurology, Gailtal-Klinik Hermagor, Hermagor, Austria; 19 Department of Neurology, Allgemeines Krankenhaus der Stadt Linz, Linz, Austria; 20 Department of Neurology, Landesklinikum St. Pölten, St. Pölten, Austria; 21 Department of Neurology, Landeskrankenhaus Rankweil, Rankweil, Austria; 22 Department of Neurology, Landeskrankenhaus Judenburg Knittelfeld, Knittelfeld, Austria; 23 Department of Neurology, Landeskrankenhaus Vöcklabruck, Vöcklabruck, Austria; 24 Department of Neurology, Sozialmedizinisches Zentrum Süd Kaiser-Franz-Josef Spital, Vienna, Austria; Friedrich-Alexander University Erlangen, Germany

## Abstract

**Background:**

In 2008 the Austrian Task Force for Neuromyelitis Optica (NMO) started a nation-wide network for information exchange and multi-centre collaboration. Their aim was to detect all patients with NMO or NMO spectrum disorders (NMO-SD) in Austria and to analyse their disease courses and response to treatment.

**Methods:**

(1) As of March 2008, 1957 serum samples (of 1557 patients) have been tested with an established cell based immunofluorescence aquaporin-4 antibody (AQP4-ab) assay with a high sensitivity and specificity (both >95%). All tests were performed in a single reference laboratory (Clinical Dept. of Neurology of the Innsbruck Medical University). (2) A nation-wide survey with several calls for participation (via email newsletters, articles in the official journal of the Austrian Society of Neurology, and workshops) was initiated in 2008. All collected data will be presented in a way that allows that every individual patient can be traced back in order to ensure transparency and to avoid any data distortion in future meta-analyses. The careful and detailed presentation allows the visualization and comparison of the different disease courses in real time span. Failure and response to treatment are made visible at one glance. Database closure was 31 December 2011. All co-operators were offered co-authorship.

**Results:**

All 71 NMO- or NMO-SD patients with AQP4-ab positivity (age range 12.3 to 79.6 years) were analysed in detail. Sex ratio (m:f = 1:7) and the proportion of patients without oligoclonal bands in cerebrospinal fluid (86.6%) were in line with previously published results. All identified patients were Caucasians.

**Conclusions:**

A nationwide collaboration amongst Austrian neurologists with good network communications made it possible to establish a database of 71 AQP4-ab positive patients with NMO/NMO-SD. This database is presented in detail and provides the basis for further studies and international cooperation in order to investigate this rare disease.

## Introduction

Epidemiological studies with detailed analysis of incidence, prevalence, natural history, risk factors and prognoses provide valuable contributions for the clinical understanding and research of diseases. From an epidemiologist’s point of view, a complete assessment of a population would be the gold standard but this goal is usually unrealistic in practice. However, a detailed epidemiological survey should be possible (a) if the respective disease causes severe symptoms that necessitate medical treatment, (b) if the respective disease is easy to diagnose and well characterized by stringent and objectified criteria, (c) if the population size is large enough to draw epidemiological conclusions, and (d) if the data collection is comprehensive and complete to the best knowledge of the investigator. To avoid any selection or reporting bias, emphasis must be put on the validity of the data collection from the diagnosis up to the entry of the data into a national data base [1]. An epidemiological study of neuromyelitis optica (NMO) in Austria, allowing for a rather small population in Central Europe, should meet these demands.

Firstly, NMO is a rare, idiopathic relapsing demyelinating disease of the central nervous system (CNS) which is easy to diagnose in the majority of cases through well-established diagnostic criteria [2-5]. But it should always be kept in mind that every neurologist, who treats patients with MS or NMO, has some examples that are a difficult diagnostic challenge. In addition, the presence of highly specific serum autoantibodies against the AQP4 water channel supports the diagnosis of clinical definite NMO and allows an early diagnosis of NMO, even if the clinical criteria are not yet fulfilled. This is the case in NMO spectrum disorders (NMO-SD) such as recurrent optic neuritis or longitudinally extensive transverse myelitis (LETM). 

Secondly, Austria, with its small population of 8.4 million people, provides an area-wide good medical supply system. In addition, good network communication is provided by the Austrian Society of Neurology and by the National Task Force for NMO (‘ARGE NMO’ [ARbeitsGEmeinschaft NeuroMyelitis Optica], http://www.i-med.ac.at/neurologie/ARGE_NMO/Index.html). A nationwide collaboration was initiated by ARGE NMO and has already resulted in the publication of several studies [6-11]. The vigilance of Austrian neurologists for NMO and NMO-SD is considered to be high (see methods below).

**Figure 1:**
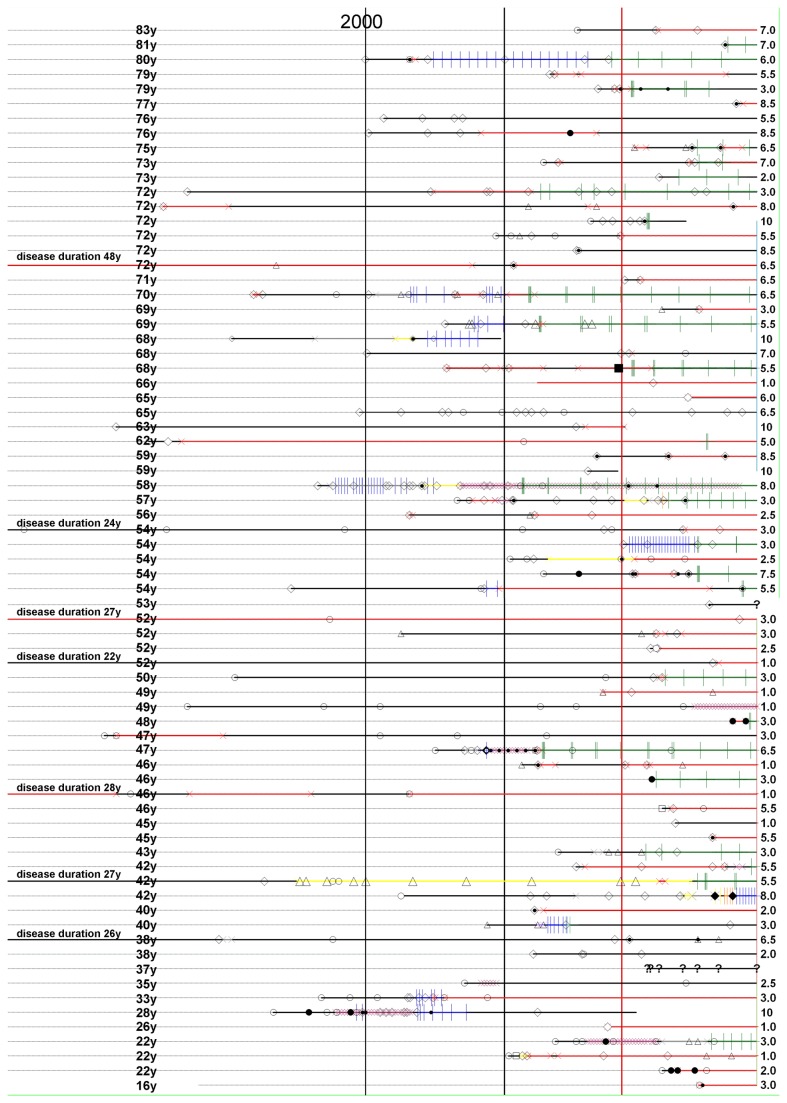
Demographic and clinical data, traceable for each individual patient. A, patients aged 16 to 53 years; B, patients aged 54 to 83 years. Note, the data of the two ‘AQP4-ab negative’ patients were also presented here in Figure 1 to ensure full transparency. However, both ‘AQP4-ab negative’ patients have no ID numbers. The ID numbers were reserved for the included ‘AQP4-ab positive’ patients. Both ‘AQP4-ab negative’ patients were clearly marked as ‘AQP4-ab negative’ and were also not included in further analyses (e.g. Figure 2). **Dashed line**, lifespan; **bold line**, disease duration; **continuous bold vertical line**, publication date of Lennon et al., Lancet Neurol 2004; **continuous dashed vertical line**, start of AQP4-Ab testing at the Innsbruck Medical University. **White circle**, unilateral optic neuritis (ON); **black circle**, bilateral ON; **square**, longitudinally transverse myelitis (LETM); **triangle**, myelitis less than 3 vertebral segments; **white rectangle** (brain lesion); **black rectangle**, tumor-like lesion; all ON and LETM (or myelitis) were treated with intravenous methylprednisolone (ivMP), unless otherwise indicated. A dot within a circle or square indicates that in addition to ivMP PLEX was performed; **a solitary black dot** indicates PLEX apart from a relapse **straight cross in green**, rituximab (RTX); **straight cross in orange**, natalizumab; **straight cross in blue**, intravenous cyclophosphamide (CTX) or mitoxantrone (MXT); **oblique cross in red**, peroral immunsuppressive therapy with azathioprine (AZA) and/or prednsiolone (PRED) and/or mycophenolate (MMF) and/or methotrexate (MTX) and/or cyclosporine A (CSA) and/or CTX; **oblique cross in purple**, intravenous immunoglobuline (IVIG); **oblique cross in grey**, interferon beta; **oblique cross in yellow**, glatiramer-acetate.

Thirdly, the same highly sensitive and specific cell-based immunofluorescence assay was used to detect AQP4-ab in the sera of all patients and, essential for quality insurance, the testing was performed *centrally*, i.e. by the same laboratory at the Clinical Department of Neurology, Innsbruck Medical University (Figure 1) [10]. The following is the result of a nationwide epidemiological study of patients with NMO/NMO-SD in Austria. With a supposed prevalence of NMO of between 0.3 and 3 people per 100,000 people in Caucasian populations [3,4,11,12], at least 25 and up to 250 NMO patients may be expected in Austria. By the end of 2011 we had identified 36 patients with NMO and 35 patients with NMO-SD; all 71 patients were seropositive for AQP4-ab. Hereby follows the individual clinical key data for each patient who met the stringent inclusion criteria. The data are presented in detail so they can be used for meta-analyses and larger epidemiological studies outside Austria.

## Materials and Methods

### Subjects

The study was approved by the local Ethics Committee (Commission of Medical Ethics of Vienna); Ethic Approval/Registration Number: EK 10-180-VK and the Ethical Committee of Innsbruck Medical University; study no. UN3041 257/4.8, 21.09.2007). Informed written consent was obtained from all patients. 

### Inclusion criteria

All patients with signs and symptoms of NMO/NMO-SD living in Austria who were found to be seropositive for AQP4-ab were included.

### AQP4-autoantibody testing

Since March 2008 the serum samples of all patients were tested in one single laboratory (Clinical Department of Neurology, Innsbruck Medical University, Austria). The highly sensitive and specific cell based immunofluorescence assay for IgG antibodies to the M23 isoform of AQP4 used in this study was previously described in detail [10]. Testing for AQP4-ab was free of charge, and neurologists were encouraged to use this service extensively, even if the diagnosis ‘NMO or NMO-SD’ seemed unlikely, e.g. patients with multiple sclerosis and confluent or multifocal spinal cord lesions with detectable oligoclonal bands (OCB), recurrent idiopathic optic neuritis with or without asymptomatic CNS lesions etc. From March 2008 to 31 December 2011 a total of 1957 serum samples of 1557 patients were analysed (1557 single investigations and 400 multiple investigations of 159 patients). 

### Data collection

To ensure maximum coverage of all NMO/NMO-SD patients in Austria a nation-wide ‘call for participation’ was made by the ARGE NMO. Over the course of nearly one year all Neurological Departments and all registered neurologists in Austria were informed about this epidemiological study and their participation was requested several times by regular email newsletters, by the official journal of the Austrian Society of Neurology (‘Neurologisch’, ISSN 2223-0629), and by personal communications. As a ‘principle of fairness’, each participant who contributed patient’s data to this study was offered co-authorship in this publication. The data based case record file included initials of the patient’s name, date of birth, sex and detailed data of their clinical course (relapses, symptoms, cerebrospinal fluid [CSF]- and MRI findings, therapy) and other paraclinical parameters such as other auto-antibodies or other concomitant diseases. Physical impairment was determined by the Expanded Disability Severity Score (EDSS) [13]. The pattern of CSF OCB was determined according to established criteria in Type 1 to 5 according to Andersson [14]. Database completion was 31 December 2011.

### Statistics

This study was retrospective and exploratory. We used descriptive and analytic statistics as previously described in detail [15,16]. Nonparametric tests (Mann-Whitney, Kolmogorov-Smirnov, Bonferroni-Holm, Chi-square, Mc Nemar) were applied (statgraphics plus 5.1).

All test results were considered significant if p-values were below 0.05. All parameters (age, sex, disease onset, disease duration (DD), relapses [ON, LETM and other] and CSF) are expressed as means, medians, minimums and maximums and standard deviation and standard error of the mean. As case numbers are rather low, the data has been presented in detail including the whole life span with all milestones of the disease (onset, relapses and treatment) to make individual disease courses comparable at one glance and so that any seasonal accumulations of relapses or differences due to age or disease duration are visible (Figure 1). 

## Results

A total of 71 patients (NMO, n=36; NMO-SD, n=35) with a median age of 55.2 (ranging from 15.7 to 83.2 years) were identified and included in our study. Mean age at onset of NMO/NMO-SD was 45.7 (ranging from 12.3 to 79.6 years).The ratio male to female was 1:7. All patients were Caucasians. The diagnosis was based on clinical, MRI, CSF analysis and testing for serum AQP4-ab which had to be positive in all patients. In a few exceptional cases, the diagnosis was also confirmed by biopsy or autopsy (Figure 1, patient numbers 24, 31, 38, 61). Only six patients with an observation period of less than 20 months had had a monophasic course so far, all other patients were relapsing. The onset attack in 29 patients was myelitis, in 38 patients ON and in 3 patients NMO (simultaneous ON and myelitis at first presentation). NMO patients aged below 50 years had ON more frequently at the onset of their disease than patients above an age of 50 years (p < 0.001). 

We calculated incidence/prevalence rates retrospectively as follows: during the period from March 2008 to December 2011 when serodiagnostic testing was centralized at the Innsbruck University Clinic, we could establish the diagnosis NMO/NMO-SD in 17 patients. We could detect 17 patients who had their first clinical symptom of NMO/NMO-SD during the period from March 2008 to December 2011 when serodiagnostic testing was centralized at the Innsbruck University Clinic. Thus we found an annual incidence rate of 0.054 per 100,000 (95% confidence interval (CI), 0.01-0.31). At the end of the observation period 4 patients had died and 5 patients had been lost to follow up. In none of the 4 deaths, NMO could be proved as a cause of death. Thus, we found by the end of 2011 a prevalence rate of 0.71 (0.77 when including the lost follow-ups) cases per 100,000 (CI, 0.17 - 0.96). 

**Figure 2:**
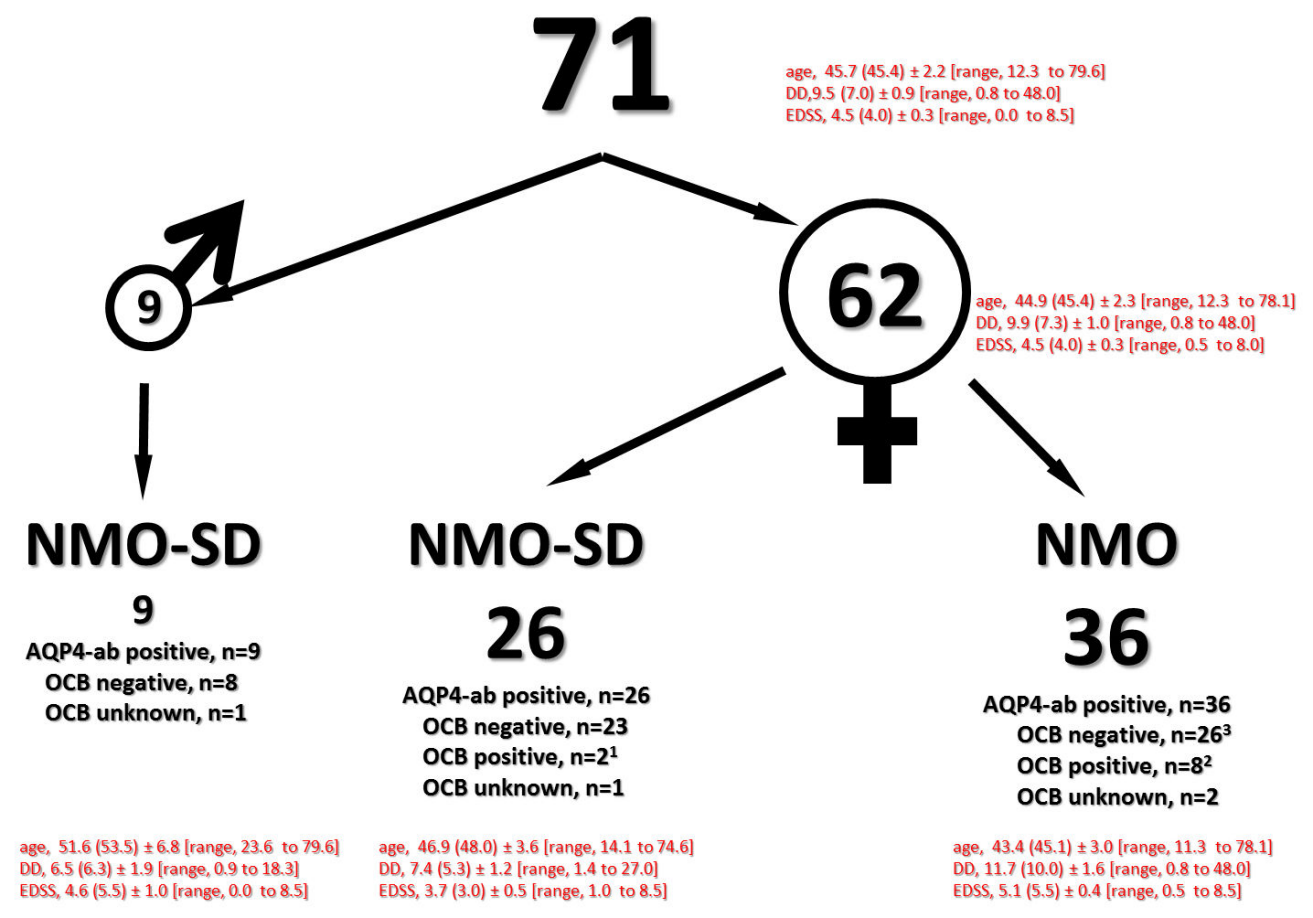
Descriptive epidemiological data, summary. OCB typing according to established criteria (type 1 - 5, Andersson et al., 1994; [12]). **1**, OCB - type 3; **2**, OCB - type 2 and 3; **3**, in 3 NMO patients OCB changed (from negative to positive (types 2 and 3)).

A summary of detailed demographic and clinical data for each NMO patient is given in Figures 1 and 2. 

Brain MRI at diagnosis was negative in 54 out of 71 NMO/NMO-SD patients (76.1%). Unspecific MRI lesions in the brain were found in 11 NMO/NMO-SD patients (15.5%; NMO, n=5 and NMO-SD, n=6). (Figure 1, patient numbers 12, 16, 23, 25, 30, 34, 37, 42, 49, 59 and 63). Five patients (7.1%; NMO, n=3 and NMO-SD, n=2) (Figure 1, patient numbers, 57, 62, 68, 70, 71) fulfilled the Barkhof criteria for MS. A cerebral tumour-like lesion (biopsy verified) was found in one patient (Figure 1, patient number 48; reference [17]; manuscript in preparation.). 

**Figure 3:**
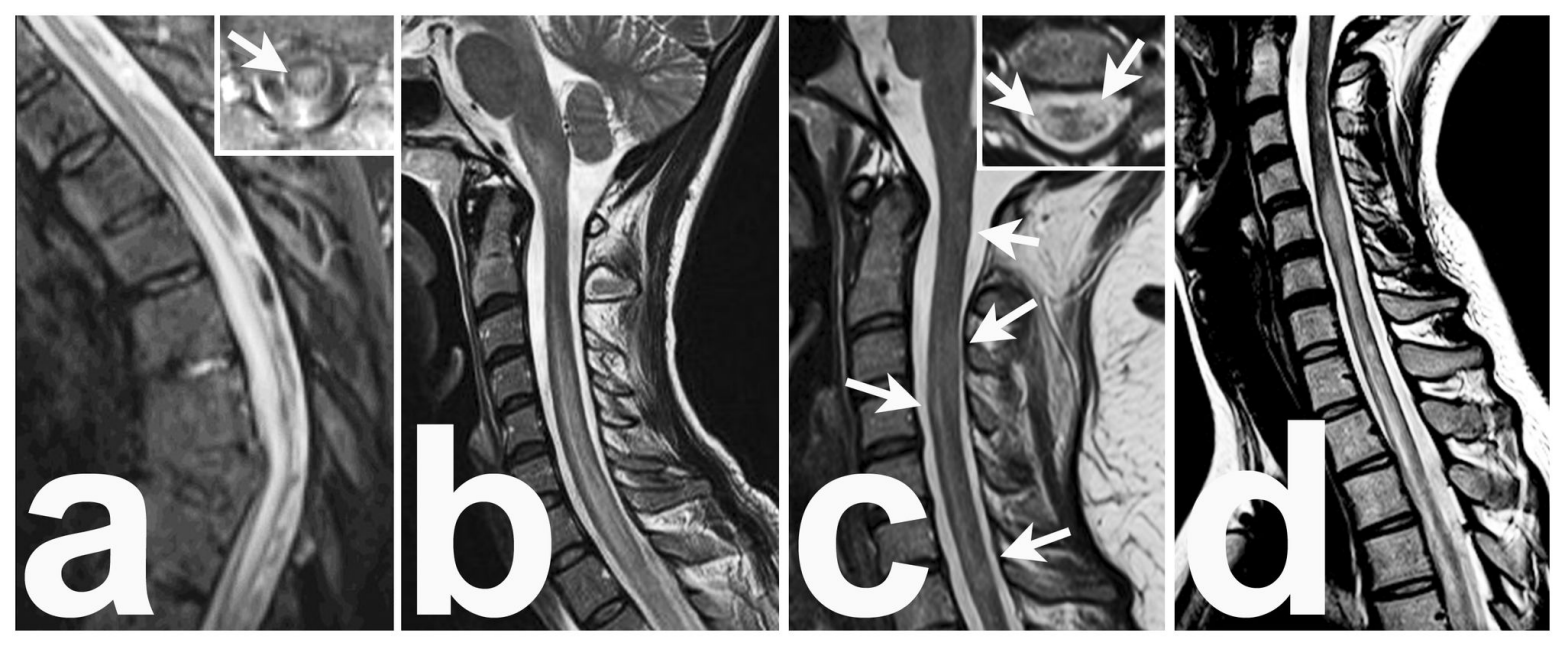
Differential diagnosis of NMO/NMO-SD associated LETM. **a**, a 90 year-old woman with left-sided chest pain, a three-day progressive paraparesis and Herpes simplex lesions gluteal. Inlay, the lesion involves the central regions of the spinal cord. CSF, 160 cells/μl (lymphocytes, (activated) lymphocytes, monocytes, neutrophils, eosinophils), no intrathecal Ig synthesis and no oligoclonal bands. Sera AQP4-ab negative. Diagnosis, HZV myelitis sine herpete (PCR from the vesicle contents, positive for HSV; PCR from CSF, negative for herpes simplex virus, but positive for varicella zoster virus.) . **b**, a 34 year old woman with peracute tetraparesis after infection of the upper respiratory tract and pneumonia. CSF, 65 cells/μl, elevated total protein and IgG concentration, positive OCB. Serum AQP4-ab were negative. Diagnosis, postinfectious myelitis (complement binding reaction and ELISA for mycoplasma pneumoniae were both positive). In a follow-up after 6 months the AQP4-ab were again negative. **c**, a 27 year-old woman with dysaesthesia and right-sided hemiparesis. MRI, several confluent lesions which are located at the lateral and posterior regions of the spinal cord. CSF, 9 cells/µl, intrathecal IgG synthesis and oligoclonal bands type 2b. Serum AQP4-ab were negative. Diagnosis, relapsing-remitting multiple sclerosis (Barkhof criteria fulfilled). **D**, NMO associated LETM, a 19 year-old women; CSF (3times), elevated cell counts, 9/µl, 181/µl and 207/µl, elevated total protein, but no IgG synthesis and no OCB. AQP4-ab were repeatedly positive (patient 71, Figure 2).

OCB were negative in 57 out of 67 NMO/NMO-SD patients (85%) (Figure 3). For 4 patients there were no data available on OCB (NMO, n=2; NMO-SD, n=2) (Figure 1, patient numbers 12, 41, 53, 56). OCB (type 2 or 3) were positive in 10 NMO/NMO-SD (12.7%; NMO, n=8 and NMO-SD, n=2 (Figure 1, patient numbers 2, 19, 27, 29, 31, 32, 57, 60, 67, 70). In 4 (out of 67 patients) who had more than one CSF examination a switch from OCB positive to OCB negative or vice versa could be observed (Figure 1, patient numbers 15, 22, 23, 37).

Interestingly, there were no male patients with NMO according to the revised Wingerchuk criteria in our study group. All men included in our study belonged to the NMO-SD group (n=10). They had either only LETM (n=8) (Figure 1, patient numbers 2, 21, 22, 53, 56, 61, 64, 69) or ON (n=1) (Figure 1, patient number 49). Most importantly, they all had a normal brain MRI and negative CSF OCB (type 1, reference 14), (Figure 1, patient numbers 21, 22, 49, 53, 56, 61, 64, 69, and Figure 2). Concomitant diseases accounted for: autoimmune thyroiditis (Figure 1, patient numbers 19, 34), autoimmune haemolytic anaemia (Figure 1, patient numbers 25, 34), systemic lupus erythematodes (Figure 1, patient numbers 12, 34, 41, 42, 62), sarcoidosis (Figure 1, patient number 55), myasthenia gravis (Figure 1, patient numbers 20, 67), celiac disease (Figure 1, patient number 58), Sjoegren syndrome (Figure 2, patient number 62), mamma carcinoma (Figure 1, patient number 67), neuroendocrine pancreas carcinoma (Figure 1, patient number 1), late onset type I diabetes mellitus (Figure 1, patient number 20) and acute hearing loss (Figure 1, patient numbers 9, 54). All were female patients. 

Other detectable serum-autoantibodies included: antinuclear antibodies (Figure 1, patient numbers 12, 19, 24, 34, 39, 42, 64, 71), anti-neutrophil cytoplasmatic antibodies (Figure 1, patient number 19), anti-double-stranded DNA (Figure 1, patient number 19), anti-Ro antibodies (Figure 1, patient number 9), anti-histone antibodies (Figure 1, patient number 29), anti-cardiolipid antibodies and anti-beta2-glycoprotein antibodies (Figure 1, patient numbers 11, 39), anti-mitochondrial antibodies (Figure 1, patient number 39), antiparietal cell antibodies (Figure 1, patient number 39) and anti-GAD antibodies (Figure 1, patient number 20). All were female patients.

## Discussion

It is intensely discussed whether NMO and NMO-SD is an own disease, or a subform of multiple sclerosis (MS), but undisputable is that the primary cause of NMO and NMO-SD is unknown. The diagnosis of NMO and NMO-SD is based on established stringent criteria, and most importantly, the clinical presentation must not be explained better by another disease or syndrome [2-5]. The diagnosis must be checked in regular intervals. 

As the overwhelming number of patients with established clinical NMO/NMO-SD diagnosis was seropositive for AQP4-ab (71 NMO/NMO-SD seropositive vs. 2 seronegative NMO-SD patients, one male with one episode of LETM, and one female with recurrent ON), we decided to include only seropositive NMO/NMO-SD patients. However, the data of the 2 ‘AQP4-ab negative’ patients were presented to allow the readers their own interpretation (Figure 1).

 Of course, we might have missed a few patients with NMO-SD, even though a highly sensitive and specific AQP4-ab immunoassay [10] was used, but this may be due to the fact (1) that AQP4-ab titres were below the detection threshold or (2) that AQP4-ab were presented as isoforms, which escaped detection, or (3) that the diagnosis of NMO/NMO-SD has yet not been established in few patients. It remains unclear how many patients with severe disabilities in nursing homes and rehabilitation clinics could not be detected. Neither sera nor data were sent to Innsbruck patients from nursing homes. 

Our data was conclusive when compared to previous observations [2,18-22]. (1) NMO/NMO-SD is a rare demyelinating disease. Taking into account the time of the first symptom of NMO/NMO-SD and not the time of diagnosis, we calculated an annual incidence rate of 0.054. The prevalence rate was 0.71, which is lower than in the two other European epidemiological studies published so far (4.4 and 2.0 per 100,000) [19,20]. This may be partly due to our stringent inclusion criteria. However, with regard to our incidence rate, it would appear that we have not identified all patients with currently ongoing NMO/NMO-SD. Thus the prevalence rate of 0.71 per 100,000 seems to be too low. The ratio of patients with NMO/NMO-DS to patients with multiple sclerosis (MS) is estimated to range from 1:1000 to 1:100 with regard to the population investigated [3,4,11,12]. In our study, this ratio was about 1:170 (a total of 71 NMO or NMO-SD patients in a population of 8.4 million, and an MS prevalence of 149 per 100,000 in 2011 (source, Austrian MS society)). This high ratio is surprising when one takes into consideration that all our patients were Caucasians and that demonstration of AQP4-ab was essential in our stringent diagnostic criteria for NMO/NMO-SD.

(2) NMO/NMO-SD affects women 5 to 9 times more frequently than men. In our study, the factor was 7 to 1 (62 female to 9 male patients). The disease course was recurrent in 87% of the patients and in those where it was monophasic the observation period was less than 20 months, therefore too short to draw a definite conclusion. All patients with concomitant additional autoimmune disorders or with other autoantibodies were females. The rate of concomitant SLE was 7%, which is higher than in the literature [23]. However, this was not the case for concomitant myasthenia gravis (3%) [24,25]. 

(3) We could also detect disorders which recently were new or had found to be been recently associated with NMO such as acute hearing-impairment [26] in 3% of our patients or late onset type I diabetes mellitus with anti-GAD antibodies in one patient (1.5%) [27]. 

(4) Unlike in MS, where the disease in general manifests itself in young adults, in NMO/NMO-SD there are no peaks. NMO/NMO-SD manifests itself in young and old [2-5]. In 40 out of 71 NMO or NMO-SD patients in our study the disease started at the age of 50 or above. This is consistent with previously published data (e.g. [2-5]).

(5) NMO/NMO-SD very rarely follows a progressive disease course [28]. In general, physical impairment develops stepwise by accumulation of several successively, temporarily well definable focal defects. Also in our study, ‘EDSS deterioration’ was caused by new relapses with well-defined focal lesions. We found no chronic disease progression in NMO or NMO-SD patients, even in those patients with a very long history of the disease. On the contrary, in some patients we observed that even very large NMO/NMO-SD lesions in the spinal cord (e.g. patient 71) or brain (e.g. patient 48) disappeared almost completely in MRI, and that the remaining lesions left only subtle clinical sequelae or even recovered completely. The significance of such NMO or NMO-SD lesions remains unclear. 

(6) The disease started in 37 patients as LETM, with a lesion extension over 3 or more vertebral segments in T2 weighted MRI images. Although the presence of LETM is a hallmark for NMO/NMO-SD, one has to be aware, that LETM may also occur in various other inflammatory or autoimmune disorders [29,30] (Figure 3). Furthermore, spinal cord lesions that are composed of many, but confluent, single lesions may appear as one single lesion extending over 3 or more vertebral segments in patients with MS, and must not be confused with non-acute LETM lesions (Figure 3). The definition of LETM as it is used for diagnostic criteria should be exclusively used for the acute stage of myelitis. Demonstration of AQP4 seropositivity in a patient with the first LETM is reliable proof of the first manifestation of NMO/NMO-SD. In cases of LETM and negative AQP4-ab status, a careful analysis of MRI- and CSF findings, as well as exclusion of other disorders causing LETM, point to the correct diagnosis [2-4,8,29-33]. To quote Matthews et al. [33] ”To truly characterize the lesion distribution of patients with NMOSD, it has been necessary to only include AQP4-ab–positive patients in our study cohort, i.e., so that the diagnosis is not in doubt. (Neurology 2013, 80: p1336). 

(7) Interestingly, the brain MRI data of our study were similar to some [20,34], but significantly different to others [19,22,33,35]. These overt discrepancies of brain lesion detection rates may be explained by pathogenetic, yet not defined mechanisms or the different study design of epidemiological or specific MRI-studies, which either may be prospective, or retrospective and exploratory, but in any case, they are exploratory. Due to the use of standardized study MRI protocols more sensitive for the white matter [36] and MR devices with higher field strength in future, the detection rate of brain lesions increases per se [37].

(8) CSF findings in NMO or NMO-SD differ markedly from MS patients. In general, the cell counts and total protein values are higher than in MS patients, while OCB are only rarely positive (in less than one-fifth of the study population [2,32]). In our study CSF of nearly all NMO/NMO-SD patients (69 out of 71) was analysed and confirmed these findings. OCB were negative in 85% of rateable CSF samples (58 out of 67 patients) (Figure 2). 

In conclusion, we established a nation-wide network for information exchange and multi-centre collaboration, and we offered the testing for AQP4-ab with a highly sensitive and specific cell-based AQP4-ab immunoassay in one single reference laboratory [10]. The testing for AQP4-ab was free of charge, and Austrian neurologists were encouraged to use this service extensively (1) to screen a large proportion of neurological patients with CNS lesions and (2) to collect experience with the test. This guaranteed that the already high level of clinical vigilance has been further increased, and suggested that the majority of Austrian patients with NMO/NMO-SD has been covered by our study.
